# LncRNA DHRS4-AS1 Inhibits the Stemness of NSCLC Cells by Sponging miR-224-3p and Upregulating TP53 and TET1

**DOI:** 10.3389/fcell.2020.585251

**Published:** 2020-12-23

**Authors:** Fei Yan, Wei Zhao, Xiaoyue Xu, Chenchen Li, Xiaoyou Li, Siwen Liu, Lin Shi, Yuan Wu

**Affiliations:** ^1^Jiangsu Cancer Hospital, Jiangsu Institute of Cancer Research, The Affiliated Cancer Hospital of Nanjing Medical University, Nanjing, China; ^2^School of Laboratory Medicine/Sichuan Provincial Engineering Laboratory for Prevention and Control Technology of Veterinary Drug Residue in Animal-Origin Food, Chengdu Medical College, Chengdu, China

**Keywords:** non-small cell lung cancer, DHRS4-AS1, miR-224-3p, TP53, TET1, cancer cell stemness

## Abstract

Non-small cell lung cancer (NSCLC) is the leading cause of cancer-related death. This study aimed to examine the roles of DHRS4-AS1/miR-224-3p signaling in the cancer cell stemness of NSCLC. Real-time PCR showed that DHRS4-AS1 was downregulated in cancerous tissues, and bioinformatics analysis revealed that high DHRS4-AS1 expression indicated a good prognosis for NSCLC patients. Sphere and colony formation assays showed that DHRS4-AS1 overexpression significantly suppressed NSCLC cell colony formation and stem cell-like properties. DHRS4-AS1 also abrogated the expression of OCT4, SOX2, CD34, and CD133, markedly inhibited the expression of epithelial-mesenchymal transition (EMT)-related factors, N-cadherin, ZEB1, and Vimentin, and increased E-cadherin expression in spheres. Furthermore, luciferase reporter assays and real-time PCR analysis demonstrated that DHRS4-AS1 and miR-224-3p were antagonistically repressed in NSCLC cells. RNA immunoprecipitation (RIP) analysis revealed that DHRS4-AS1 interacted with miR-224-3p. DHRS4-AS1 partially reversed the miR-224-3p-decreased TP53 and TET1, resulting in the inhibition of tumor growth *in vivo*. Finally, TP53 and TET1 were antagonistically regulated by DHRS4-AS1 and miR-224-3p in NSCLC cells. In conclusion, TP53- and TET1-associated DHRS4-AS1/miR-224-3p axis is an essential mechanism by which NSCLC modulates cancer cell stemness.

## Introduction

Lung cancer is the most common cancer worldwide and ultimately causes the death of many patients (Chen et al., 2016; Siegel et al., 2020). Non-small cell lung cancer (NSCLC) is a common type of lung cancer, accounting for up to 85% of all lung cancer cases, and the 5-year survival rate of NSCLC patients remains poor (Boolell et al., 2015). Although effective treatment has been achieved for NSCLC patients in the clinic, most cancers recur after surgery or radiotherapy (Wood et al., 2015; Duma et al., 2019). The molecular mechanisms of NSCLC have not been fully explored. Undoubtedly, the identification of biomarkers for the prognosis and treatment of patients with NSCLC is urgently needed. To produce better therapeutic strategies, it is essential to elucidate the signaling pathways of metastasis and recurrence of NSCLC progression. The recurrence of NSCLC is associated with cancer stem cells. Cancer stem cells are potentially responsible for cancer cell self-renewal, initiation, proliferation, and differentiation, can produce aggressive and metastatic tumors, as well as multidrug resistance (Kusoglu and Biray Avci, 2019).

Long non-coding RNAs (lncRNAs) were reported to contribute the initiation and development of cancers by our and other groups (Bautista et al., 2018; Chang et al., 2018; Zhao et al., 2018; Huang et al., 2019; Li et al., 2020). HOX transcript antisense RNA (HOTAIR) enhances lung cancer cell invasion and motility (Zhao et al., 2014a). Maternally expressed 3 (MEG3) inhibits cancer cell proliferation and increases p53-mediated cancer cell apoptosis during NSCLC (Lu et al., 2013). Metastasis-associated lung adenocarcinoma transcript 1 (MALAT1) is considered a key marker of metastasis in lung cancer (Gutschner et al., 2013). The lncRNA actin filament-associated protein 1 antisense RNA 1 (AFAP1-AS1) promotes NSCLC tumorigenesis and chemoresistance (Yin et al., 2018; Huang et al., 2019). SBF2-AS1, an oncogenic lncRNA, increases NSCLC cell proliferation (Lv et al., 2016). Most importantly, lncRNAs also play roles in the stemness of lung cancer. For instance, the long non-coding RNA Linc00662 promotes cell invasion and contributes to cancer stem cell-like phenotypes in lung cancer cells (Gong et al., 2018). The lncRNA FENDRR suppresses cancer cell stemness by downregulating multidrug resistance gene 1 (MDR1) expression by competitively binding with HuR, an RNA-binding protein that plays a role in NSCLC progression (Gong et al., 2019). The lncRNA CASC11 increases TGF-beta1-mediated cancer cell stemness, resulting in a poor overall survival rate for NSCLC patients (Fu et al., 2019). The lncRNA linc-ITGB1 suppresses cancer stemness by inhibiting Snail expression in NSCLC (Guo et al., 2019). The lncRNA HAND2-AS1 inhibits cancer cell migration and invasion and maintains cancer cell stemness by interacting with TGF-beta1 (Miao et al., 2019). According to the databases “Gene Expression Profiling Interactive Analysis (GEPIA, http://gepia.cancer-pku.cn/index.html)” data, DHRS4-AS1 might be an important lncRNA in lung cancer progression. Although the function of DHRS4-AS1 was characterized in other cancers (Wang et al., 2018; Luan et al., 2019; Utnes et al., 2019), the role of DHRS4-AS1 in NSCLC still needs uncovery. Our study revealed that the expression of DHRS4-AS1 in tumors correlates with the overall survival of NSCLC patients, and DHRS4-AS1 functions as a tumor suppressor by regulating cancer cell colony formation and stemness. We conclude that TP53- and TET1-associated DHRS4-AS1/miR-224-3p signaling plays important roles in NSCLC progression *in vitro* and *in vivo*. Consequently, DHRS4-AS1 and miR-224-3p are promising therapeutic targets for NSCLC.

## Materials and Methods

### Clinical Sample Collection

Eighty-three pairs of lung cancer and adjacent tissues were collected from NSCLC patients at the Affiliated Cancer Hospital of Nanjing Medical University. None of the patients had been treated clinically before surgery. The collected tissues were immediately stored in liquid nitrogen until further analysis. Clinical specimen collection was approved by the Research Ethics Committee of the Affiliated Cancer Hospital of Nanjing Medical University (ID: 2018RC1D), and a consent form was signed by each participating patient.

### Haematoxylin-Eosin (H&E) Staining

Clinical lung tissues were fixed in 4% paraformaldehyde and subjected to sectioning. H&E staining was performed as previously described (Zhao et al., 2014b). All chemicals and agents used were purchased from Sigma (Shanghai, China).

### Cell Culture, Transfection, and Infection

Human lung cell lines (BEAS-2B, A549, PC-9, Calu-3, SPCA-1, and H1299) were purchased from China Infrastructure of Cell Line Resource (Beijing, China). All cell lines were maintained in RPMI 1640 basic medium with 10% fatal bovine serum (FBS) (Gibco, Thermo Fisher Scientific, Shanghai, China). Cells were placed in a 37°C humidified incubator with 5% CO_2_. The medium was supplemented with 10% fetal bovine serum (FBS), 100 mg/mL streptomycin, and 100 U/mL penicillin (Gibco).

In this study siRNAs was synthesized by GenePharma (Shanghai, China) and transfected by using Lipofectamine-3000 (Invitrogen, Thermo Fisher Scientific) according to the manufacturer's instructions. The stable cells were generated by letivirus infection, which contains shRNAs and plasmids (GenePharma). Scramble, 5′-UCUGCGAACGUCUCACGUU-3′; miR-224-3p (mimic): 5′-AAAAUGGUGCCCUAGUGACUACA-3′; anti-miR-224-3p (inhibitor), 5′-UGUAGUCACUAGGGCACCAUUUU-3′; si-NC, 5′-AGA UCG ACG UGG CGU AAU CCA-3′; si-DHRS4-AS1, 5′-GGCCUACAAUACGAAUAAAUU-3′. Additionally, the full length of DHRS4-AS1 (ID: ENSG00000215256) was constructed into virus DNA by GenePharma. The cancer cell lines were infected with lentivirus according to the manufacturer's instructions.

### Sphere and Colony Formation of Cancer Stem Cells

Cancer stem cells were sorted from A549 and Calu-3 cells using spherocyte medium. In total, 2 × 10^4^ cells were plated onto six-well ultra-low cluster plates (NUNC, Thermo Fisher Scientific) and cultured in DMEM/F12 serum-free medium (Gibco) supplemented with epidermal growth factor (20 ng/mL), b-fibroblast growth factor (20 ng/mL), insulin (4 μg/mL), and B27 (2%) (all from Sigma). The number of spheres was captured and counted under an inverted microscope (Leica, Oskar-Barnack-Straße, Germany) after 10 days. Cancer stem cells were identified by western blot or real-time PCR analysis of the expression of stem cell markers, including OCT4, SOX2, CD34, and CD133.

Cancer stem cells were separated using CD133-binding magnetic bead kit (Becton, Dickinson and Company, New Jersey, USA), and 2 × 10^4^ stem cells were then plated into six-well plates with an ultra-low attachment surface (MUNC). These cells were maintained in sphere formation medium for 7 days, and the indicated siRNAs or plasmids were transfected into sphere-forming cells using Lipofectamine 3000. Finally, the number of spheres was quantified under a microscope after an additional 20 days.

### Real-Time PCR Analysis

Total RNA was isolated from tissues and cells by using TRIzol reagent (Thermo Fisher Scientific). Then, 1 μg of RNA was used for reverse transcription in a volume of 10 μL to produce complementary DNA (cDNA) using the PrimeScript RT Kit (TaKaRa, Dalian, China). Particularly, the miRNA RT procedure, and briefly, the reactions were mixed with the total RNA, RT-PCR primer of miRNAs, PrimeScript^®^RT Enzyme Mix I, PrimeScript^®^Buffer2. These reactions were incubated in Takara PCR thermocycler for 30 min at 16°C, 30 min at 42°C, 5 min at 85°C, and then held at 4°C. Next, SYBR Premix Ex Taq II was used for real-time PCR analysis (Perfect Real Time, TaKaRa) to investigate the levels of lncRNAs, miRNAs, and mRNAs. The expression levels of lncRNAs and mRNAs were normalized to those of 18S RNA and glyceraldehyde-3-phosphate dehydrogenase (GAPDH), respectively, while the expression levels of miRNAs were normalized to those of U6. The expression levels of DHRS4-AS1 and other mRNAs were normalized to those of 18S RNA and GAPDH, respectively, and the expression levels of miRNAs were normalized to those of U6. The primers used are as follows: GAPDH, 5′-CAC CCA CTC CTC CAC CTT TG-3′ (forward) and 5′-CCA CCA CCC TGT TGC TGT AG-3′ (reverse); U6 reverse transcription PCR primer, 5′-GTC GTA TCC AGT GCA GGG TCC GAG GTA TTC GCA CTG GAT ACG ACA AAT ATG-3′; U6 real-time PCR primers, 5′-TGC GGG TGC TCG CTT CGG CAG C-3′ (forward) and 5′-GTG CAG GGT CCG AGG T-3′ (reverse); 18S RNA, 5′-CGT TCT TAG TTG GTG GAG CG-3′ (forward) and 5′-CCG GAC ATC TAA GGG CAT CA-3′ (reverse); OCT4, 5′-CCT TCG CAA GCC CTC ATT TC-3′ (forward) and 5′-TAG CCA GGT CCG AGG ATC AA-3′ (reverse); SOX2, 5′-GAC AGT TAC GCG CAC ATG AA-3′ (forward) and 5′-TAG GTC TGC GAG CTG GTC AT-3′ (reverse); CD33, 5′-GCC ACC AGA GCT ATT CCC AA-3′ (forward) and 5′-GGT CTT CGC CCA GCC TTT CT-3′ (reverse); CD133, 5′-CCC CGC AGG AGT GAA TCT TT-3′ (forward) and 5′-GAA GGA CTC GTT GCT GGT GA-3′ (reverse); E-cadherin, 5′-GCT GGA CCG AGA GAG TTT CC-3′ (forward) and 5′-CGA CGT TAG CCT CGT TCT CA-3′ (reverse); N-cadherin, 5′-AGG CTT CTG GTG AAA TCG CA-3′ (forward) and 5′-AAA TCT GCA GGC TCA CTG CT-3′ (reverse); ZEB1, 5′-GAG AGG ATC ATG GCG GAT GG-3′ (forward) and 5′-TGG CAG GTC ATC CTC TGG TA-3′ (reverse); Vimentin, 5′-AAA CTT AGG GGC GCT CTT GT-3′ (forward) and 5′-TGA GGG CTC CTA GCG GTT TA-3′ (reverse); TET1, 5′-ACT CCC TGA GGT CTG TCC TG-3′ (forward) and 5′-AGG TAG GGC TGC ATG ACT TG-3′ (reverse); TP53, 5′-CCT GGA TTG GCC AGA CTG C-3′ (forward) and 5′-TTT TCA GGA AGT AGT TTC CAT AGG T-3′ (reverse); miR-224-3p reverse transcription PCR primer, 5′-GTC GTA TCC AGT GCA GGG TCC GAG GTA TTC GCA CTG GAT TGT AGC-3′ and its real-time PCR primers, 5′-aaa aTggTg ccc Tag Tg-3′ (forward) and 5′-GTG CAG GGT CCG AGG T-3′ (reverse). The relative DHRS4-AS1, miR-224-3p, and TP53 and TET1 expression levels were calculated using the 2-ΔΔCt method.

### Western Blot Analysis

Protein samples were prepared with a protease inhibitor cocktail containing RIPA lysis buffer (Beyotime, Haimen, China). The protein lysates were denatured at 98°C for 10 min. Next, samples (~50 μg) were separated by 8% sodium dodecyl sulfate-polyacrylamide gel electrophoresis (SDS-PAGE) and then transferred to 0.22-μm polyvinylidene difluoride (PVDF) membranes (Bio-Rad, Hercules, CA, USA) using the Trans-Blot^®^ Turbo™ Transfer System (Bio-Rad). The protein-containing PVDF membranes were blocked with 1.0% bovine serum albumin (BSA) diluted with PBS. The membranes were incubated with the indicated primary antibody at 4°C for 8 h and then incubated with a goat anti-mouse or goat anti-rabbit horseradish peroxidase (HRP)-conjugated secondary antibody diluted 1:40,000 (Bioworld Technology, Nanjing, China) for 60 min. The following antibodies were used: anti-TP53 (diluted 1:1,000, Sigma, cat. #: SAB4300073), anti-TET1 (diluted 1:1,000, Sigma, cat. #: HPA019032), anti-OCT4 (diluted 1:500, Bioworld Technology, cat. #: BS70993), anti-SOX2 (diluted 1:1,000, Sigma, cat. #: SAB2701800), anti-CD34 (diluted 1:1,000, Bioworld Technology, cat. #: BS90235), anti-GAPDH (diluted 1:4,000, Bioworld Technology, cat. #: MB9231), anti-CD133 (diluted 1:500, Bioworld Technology, cat. #: BS90215), E-cadherin (diluted 1:500, Sigma, cat. #: SAB4503751), anti-N-cadherin (Sigma, cat. #: SAB2702400), anti-ZEB1 (Sigma, cat. #: SAB2500097), and anti-Vimentin (Sigma, cat. #: SAB4300676). The protein bands were visualized by enhanced chemiluminescence (ECL) using Bio-Rad equipment and analyzed by Quantity One software (Bio-Rad).

### Dual-Luciferase Reporter Assays

The cancer cell lines were seeded into 24-well plates at a density of 6 × 10^4^ cells per well. The cells were co-transfected with the pmirGLO-DHRS4-AS1-WT, pmirGLO-DHRS4-AS1-mut, pmirGLO-TP53 (or TET1)-3'-UTR WT or pmirGLO-TP53 (or TET1)-3′-UTR-mut reporter plasmid and the scramble miR-224-3p mimic, or anti-miR-224-3p (inhibitor). After 24 h of transfection, luciferase activity was determined using the Dual-Luciferase Assay Kit according to the manufacturer's protocol on a GloMax 20/20 luminometer (Promega, Madison, Wisconsin, USA). In this study, the 3′-UTR sequences of isoform 1 (Transcript ID: NM_000546.6) and TET1 (ID: NM_030625) isoform 1 were obtained from the NCBI database, and the full length DHRS4-AS1 sequence (ID: ENSG00000215256) was obtained from the UCSC database. The corresponding luciferase reporter plasmids were constructed into pmirGLO empty vector by GenePharma using NheI and SbfI as restriction enzyme cutting sites for each plasmid. Three wells were utilized for each treatment group, and the experiments were repeated three times independently.

### RIP Assays

To determine the miR-224-3p binding sites on DHRS4-AS1, RIP assay was performed using the Magna RIP RNA-binding protein immunoprecipitation kit (Millipore, Massachusetts, USA) according to the manufacturer's instructions. Anti-Ago2 was diluted 1:500 (Sigma, USA, cat. #: SAB4200085). The cancer cell lysate was incubated with anti-Ago2 buffer, and IgG was used as a negative control. Immunoprecipitated RNA was then used for the real-time PCR analysis of DHRS4-AS1 and miR-224-3p. Three wells were utilized for each group.

### Animal Experiments

Stable Calu-3 cells were centrifuged at 80 g × 5 min, and 3 × 10^6^ cells were then inoculated into a 5-week-old BALB/c nude mice (Model Animal Research Center of Nanjing University, Nanjing, China). The nude mouse experiments were approved by the Research Ethics Committee of Chengdu Medical College (ID: XN19L38). Tumor volumes were calculated every 3 days for 15 days according to the following formula: tumor volume = (length × width^2^)/2. The mice were sacrificed on the 15th day after xenograft implantation.

### Statistical Analysis

The data were analyzed statistically using the SPSS software package (version 17.0, SPSS, Inc., Armonk, NY, USA) and GraphPad Prism 6 (GraphPad Software, La Jolla, CA, USA). The results are expressed as the mean ± standard deviation (SD). Statistical significance between two groups was examined by a two-tailed Student's *t*-test, and statistical significance among multiple groups was determined by using *post-hoc* analysis contrasts. *P*-values < 0.05 were considered statistically significant.

## Results

### DHRS4-AS1 Correlates With NSCLC Survival

**Figure 1:**
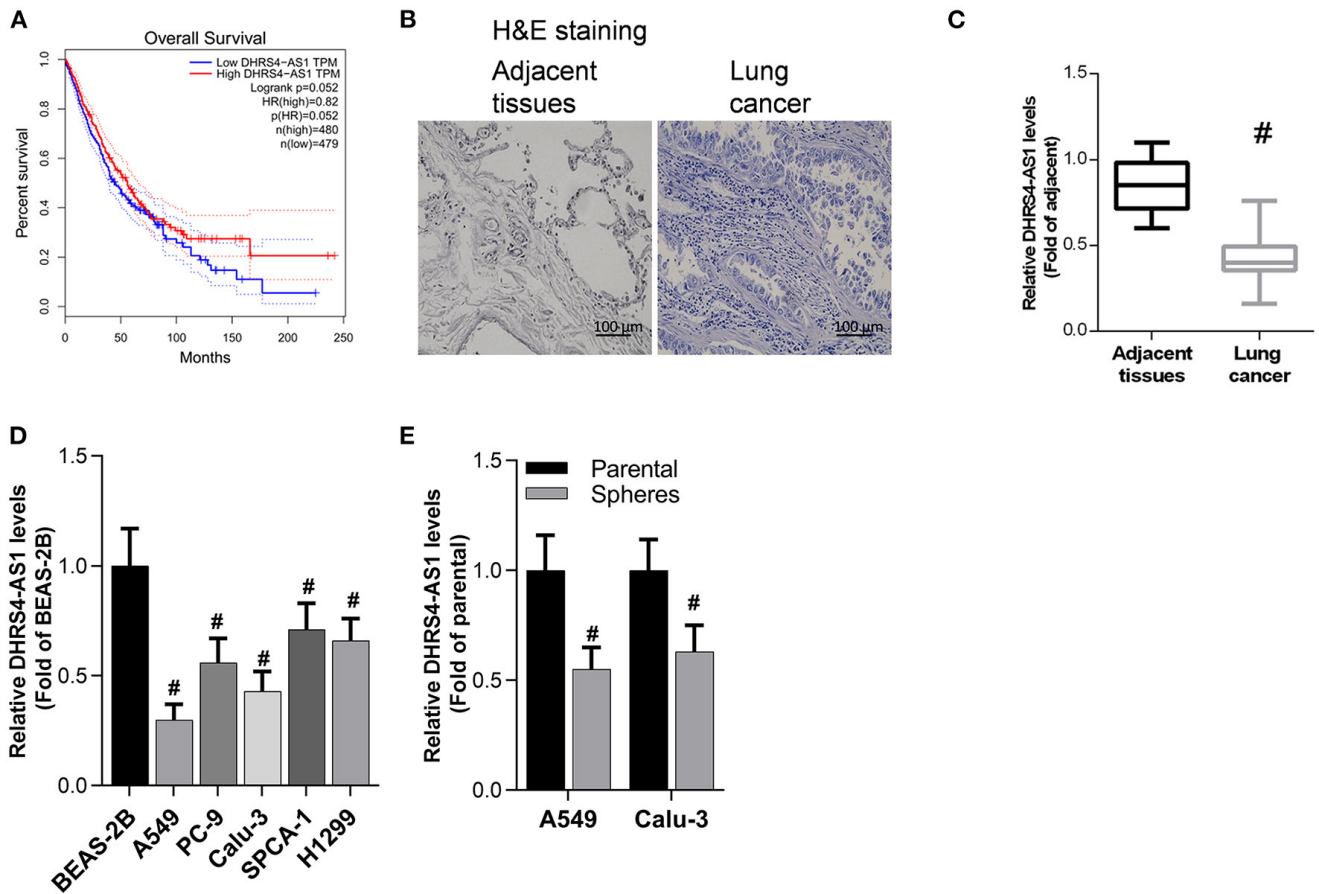
Detection of DHRS4-AS1 expression in NSCLC and its clinical significance. **(A)** The survival rate of NSCLC was analyzed on the GEPIA web portal by DHRS4-AS1 expression. **(B)** Histopathological pictures shown the adjacent normal and cancer tissues. **(C)** Real-time PCR analysis of DHRS4-AS1 expression in adjacent lung (*N* = 83) and cancer (*N* = 83) tissues; #*p* < 0.01 vs. adjacent tissues. **(D)** Real-time PCR analysis of DHRS4-AS1 expression in lung cancer cell lines and normal lung epithelial cell line; #*p* < 0.01 vs. BEAS-2B cells. **(E)** Real-time PCR analysis of DHRS4-AS1 expression in parental and sphere-forming A549 and Calu-3 cells; #*p* < 0.01 vs. parental cells. All the results are expressed as the mean ± SD from three independent experiments, and each sample was repeated in triplicate.

To explore the relationship between DHRS4-AS1 expression and NSCLC patients survival, the survival rate was analyzed in GEPIA, which base on The Cancer Genome Atlas (TCGA) and Genotype-Tissue Expression (GTEx) samples, high DHRS4-AS1 expression indicated a good overall survival outcome of patients with NSCLC (Figure 1A). Herein, we collected clinical tissues, and found that malignant proliferation of pulmonary epithelial cells in lung cancer tissues evidenced by H&E staining (Figure 1B). Real-time PCR analysis indicated that DHRS4-AS1 expression was decreased in NSCLC tissues compared with adjacent normal tissues (Figures 1B,C). In addition, DHRS4-AS1 was downregulated in lung cancer cells, including A549, PC-9, Calu-3, SPCA-1, and H1299 cells, compared with immortal human bronchial epithelial cells (BEA-2B) (Figure 1D). Importantly, real-time PCR analysis demonstrated that DHRS4-AS1 expression was lower in NSCLC cell spheres than in parental cells (Figure 1E). These findings suggest that DHRS4-AS1 is a tumor suppressor and abrogates cancer stemness in NSCLC progression.

### DHRS4-AS1 Suppresses the Colony Formation Ability and Stem Cell-Like Properties of NSCLC Cells *in vitro*

**Figure 2:**
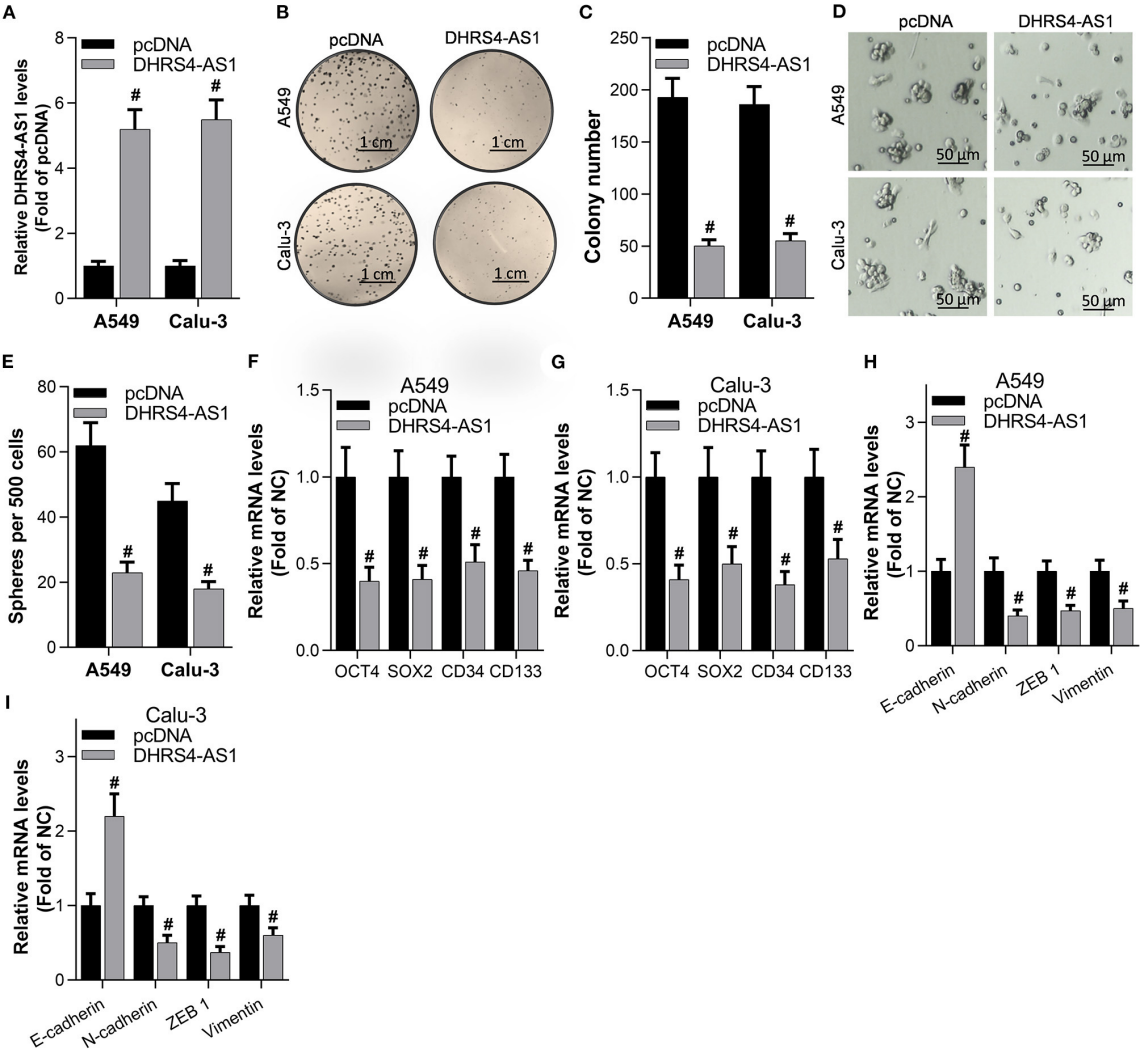
DHRS4-AS1 suppressed colony formation and stem cell-like properties *in vitro*. **(A)** Real-time PCR analysis of DHRS4-AS1 expression after 48 h of transfection in A549 and Calu-3 cancer stem cells; #*p* < 0.01 vs. pcDNA. **(B,C)** Colony formation analysis of A549- and Calu-3-derived stem cells after overexpressing DHRS4-AS1 for 10 days, at which point the colonies were captured and counted; #*p* < 0.01 vs. pcDNA. **(D,E)** Representative images of spheres formed by cancer stem cells **(D)** and quantification of cancer stem cell spheres; #*p* < 0.01 vs. pcDNA. **(F,G)** Real-time PCR analysis of the expression of cancer stemness-related genes after 72 h of transfection in A549 and Calu-3 cancer stem cells; #*p* < 0.01 vs. pcDNA. **(H,I)** Real-time PCR analysis of the expression of EMT-related factors in tumor spheres; #*p* < 0.01 vs. pcDNA. All the results are expressed as the mean ± SD from three independent experiments, and each sample was repeated in triplicate.

To determine the role of DHRS4-AS1 in modulating the cancer stemness maintenance, overexpression of DHRS4-AS1 in A549 and Calu-3 cancer stem cells (Figure 2A) significantly inhibited NSCLC cells colony formation ability (Figures 2B,C). DHRS4-AS1 also decreased sphere formation in NSCLC cancer stem cells (Figures 2D,E). Moreover, DHRS4-AS1 markedly downregulated the expression of OCT4, SOX2, CD34, and CD133, which were reported as NSCLC stemness markers (Heng et al., 2019) (Figures 2F,G and Supplementary File). Additionally, DHRS4-AS1 inhibited the expression of epithelial-mesenchymal transition (EMT)-related factors, N-cadherin, ZEB1, and Vimentin but increased E-cadherin expression in the cancer stem cell spheres (Figures 2H,I and Supplementary File).

### DHRS4-AS1 and miR-224-3p Are Inversely Repressed in Cancer Cells

**Figure 3:**
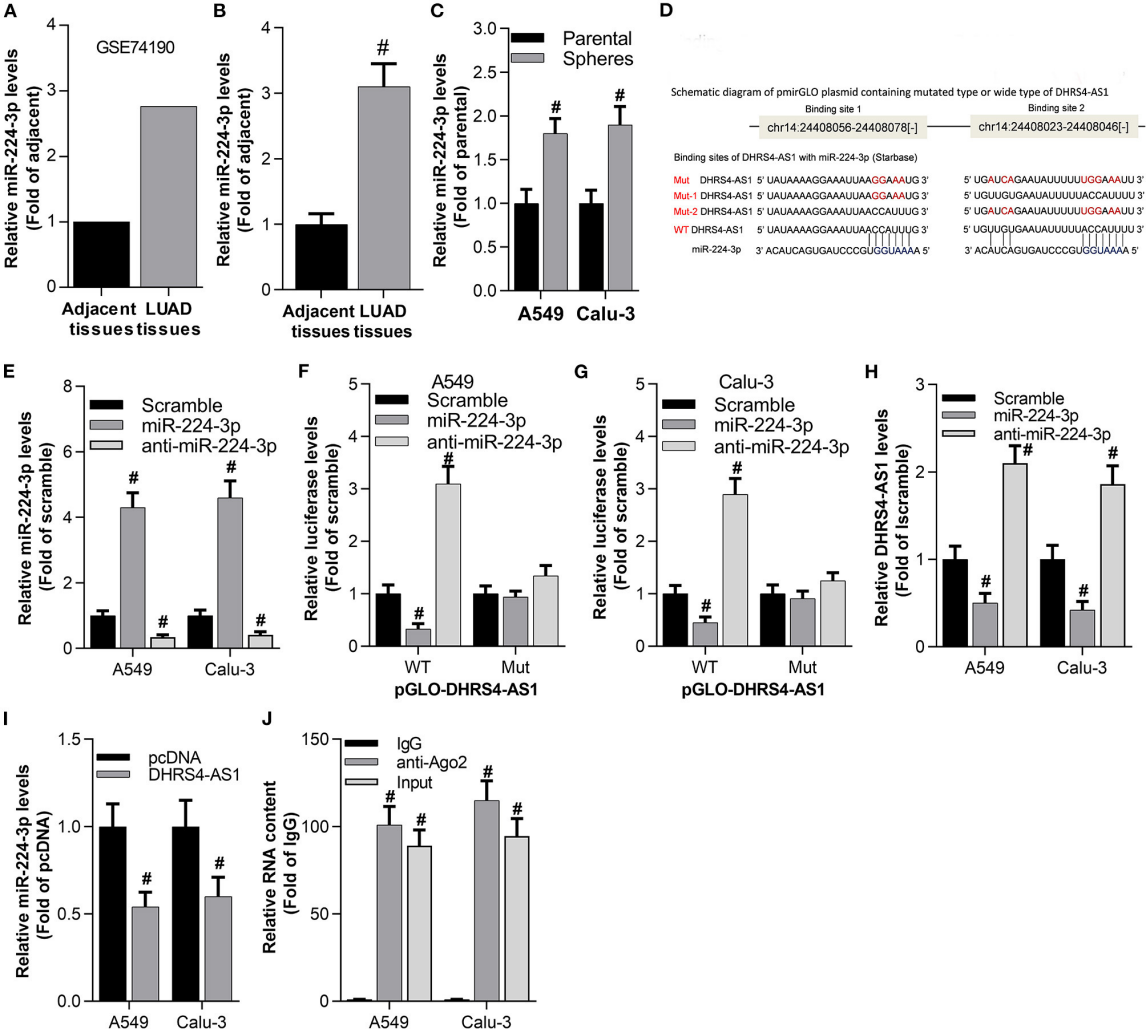
Antagonistic repression between DHRS4-AS1 and miR-224-3p in NSLC cancer cells. **(A)** Bioinformatics analysis of miR-224-3p expression in the GSE74190 dataset as determined by GEO2R analysis. **(B)** Real-time PCR analysis of miR-224-3p expression in lung cancer (*N* = 83) and adjacent (*N* = 83) tissues; #*p* < 0.01 vs. adjacent tissues. **(C)** Real-time PCR analysis of miR-224-3p expression in parental cells and A549 and Calu-3 stem cell spheres; #*p* < 0.01 vs. parental cells. **(D)** Schematic representation of the predicted miR-224-3p binding sites on the DHRS4-AS1 sequence according to the StarBase analysis. **(E)** Real-time PCR analysis of miR-224-3p expression after 24 h of transfection in A549 and Calu-3 cells; #*p* < 0.01 vs. scramble. **(F,G)** Luciferase assay showing pGLO-DHRS4-AS1-WT (wild type) and pGLO-DHRS4-AS1-Mut (Mut) after 24 h of transfection in mock-, miR-224-3p- or anti-miR-224-3p-transfected A549 and Calu-3 cells; #*p* < 0.01 vs. scramble. All data was normalized to wildtype control group (WT Scramble) **(H)** Real-time PCR analysis of DHRS4-AS1 expression after 24 h of transfection in A549 and Calu-3 cells; #*p* < 0.01 vs. scramble. **(I)** Real-time PCR analysis of miR-224-3p expression in A549 and Calu-3 cells after 24 h of DHRS4-AS1 overexpression; #*p* < 0.01 vs. pcDNA. **(J)** RIP analysis showed that DHRS4-AS1 and miR-224-3p could directly bind to AGO2 in A549 and Calu-3 cells; #*p* < 0.01 vs. IgG. All the results are expressed as the mean ± SD from three independent experiments, and each sample was repeated in triplicate.

The involved molecular mechanism of DHRS4-AS1 in NSCLC stemness was explored by bioinformatics analysis. The expression of DHRS4-AS1 and potential target miRNAs were excavated in the TCGA database. GEO2R analysis showed that miR-224-3p was upregulated in lung adenocarcinoma (LUAD, a subtype of NSCLC) (Figure 3A), which was consistent with collected NSCLC specimens (Figure 3B). Furthermore, miR-224-3p expression was higher in spheres than parental NSCLC cells (Figure 3C). Then, the binding sites of miR-224-3p and DHRS4-AS1 were predicted in StarBase (Li et al., 2014) (Figure 3D). The predicted sequence of DHRS4-AS1 (pGLO-DHRS4-AS1 WT, two miR-224-3p binding sites) and mutation (pGLO-DHRS4-AS1 Mut, two miR-224-3p binding sites were mutated) were constructed into pGLO 3′-UTR as previously reported (Clement et al., 2015). As shown here, miR-224-3p inhibited the luciferase activity of pGLO-DHRS4-AS1, while miR-224-3p knockdown increased its activity when normalized to wildtype control group (WT Scramble) (Figures 3E–G). Correspondingly, real-time PCR analysis showed that miR-224-3p inhibited DHRS4-AS1 expression in A549 and Calu-3 cells, while miR-224-3p knockout increased DHRS4-AS1 expression in these cells (Figures 3H,I). The RNA immunoprecipitation (RIP) assay revealed that DHRS4-AS1 has direct binding with miR-224-3p in cancer cells (Figure 3J). Thus, DHRS4-AS1 and miR-224-3p are inversely repressed in cancer cells.

### DHRS4-AS1 Blocks the miR-224-3p-Mediated Silencing of Colony Formation and Stem Cell-Like Properties *in vitro*

**Figure 4:**
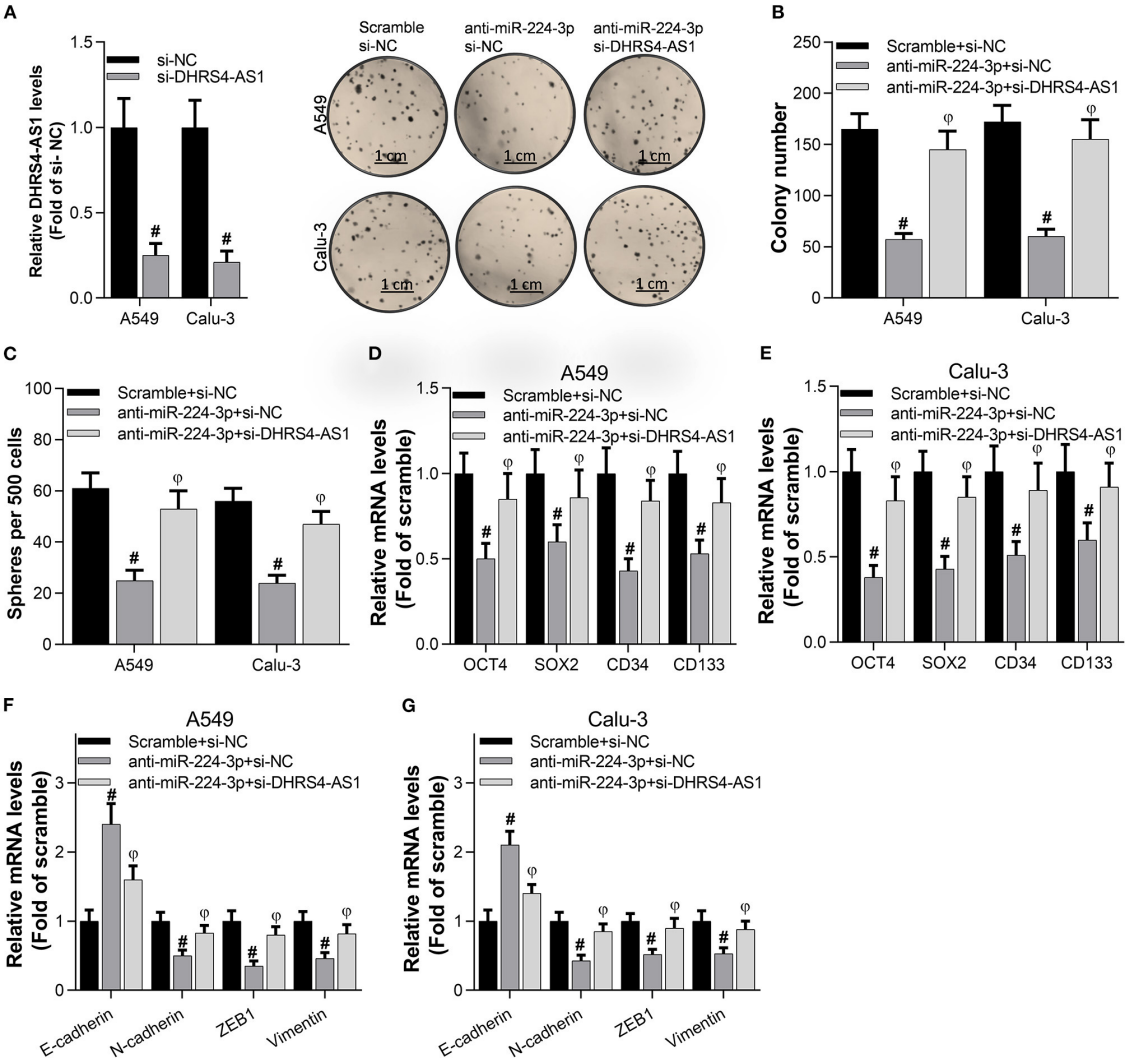
DHRS4-AS1 knockdown reversed the anti-miR-224-3p-mediated inhibition of colony formation and stem cell-like properties *in vitro*. **(A,B)** Colony formation analysis of cancer stem cell proliferation after 20 days of transfection, at which point the colonies were captured and counted; #*p* < 0.01 vs. scramble+si-NC, ^φ^*p* < 0.01 vs. anti-miR-224-3p+si-NC. And the DHRS4-AS1 levels were determined by using real-time PCR after 48 h transfection. **(C)** Quantification of cancer stem cell spheres after 72 h of transfection; #*p* < 0.01 vs. scramble+si-NC, ^φ^*p* < 0.01 vs. anti-miR-224-3p+si-NC. **(D,E)** Real-time PCR analysis of the expression of cancer stemness-related genes after 72 h of transfection; #*p* < 0.01 vs. scramble+si-NC, ^φ^*p* < 0.01 vs. anti-miR-224-3p+si-NC. **(F,G)** Real-time PCR analysis of the expression of EMT-related factors in tumor spheres; #*p* < 0.01 vs. scramble+si-NC, ^φ^*p* < 0.01 vs. anti-miR-224-3p+si-NC. All the results are expressed as the mean ± SD from three independent experiments, and each sample was repeated in triplicate.

To examine the antagonism between DHRS4-AS1 and miR-224-3p in cancer cell stemness, A549 and Calu-3 cells were transfected with anti-miR-224-3p and si-DHRS4-AS1. The data showed that interfering of DHRS4-AS1 significantly increased anti-miR-224-3p-mediated decrease in colony formation (Figures 4A,B). miR-224-3p inhibitor was proved to suppress sphere formation in A549 and Calu-3 stem cells, while sphere formation was restored by silencing DHRS4-AS1 (Figure 4C). Furthermore, miR-224-3p could markedly induce the downregulation of stemness markers, including OCT4, SOX2, CD34, and CD133; however, the suppression was reversed by knocking down DHRS4-AS1 expression (Figures 4D,E). Similarly, DHRS4-AS1 knockdown markedly reversed the silencing of the miR-224-3p-induced expression of EMT-related genes inA549 and Calu-3 cancer stem cells (Figures 4F,G). These findings reveal that the inverse regulation of DHRS4-AS1 and miR-224-3p is an essential molecular mechanism during cancer stem cell growth and the development of cancer cell stemness.

### Knockdown of DHRS4-AS1 Attenuates Anti-miR-224-3p-Mediated Tumor Growth Inhibition *in vivo*

**Figure 5:**
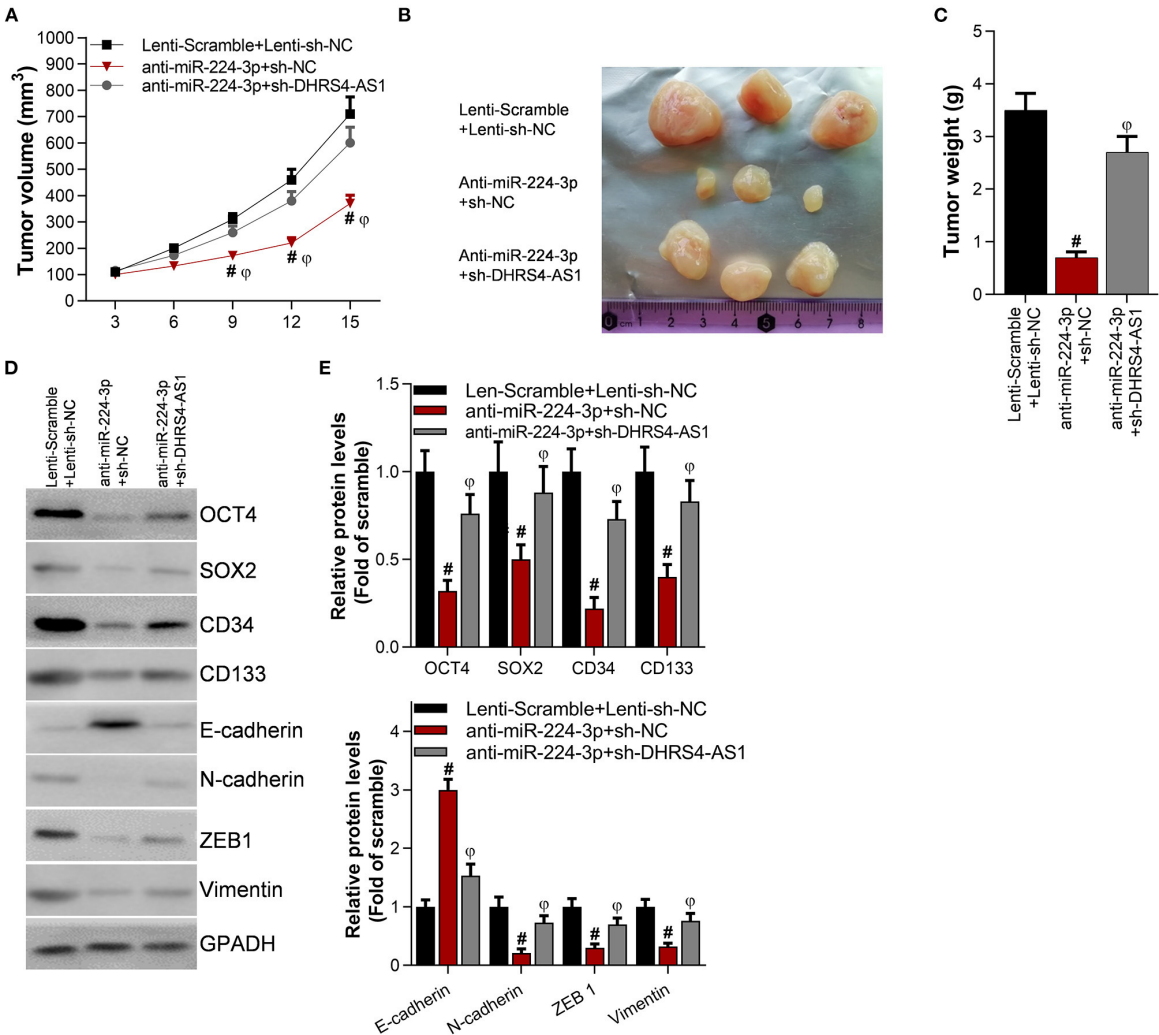
Knockdown of DHRS4-AS1 attenuated anti-miR-224-3p-mediated tumor growth inhibition *in vivo*. Calu-3 cells were transfected with lenti-viruses meidatescramble, anti-miR-224-3p, si-NC, or si-DHRS4-AS1 for 48 h. The infected Calu-3 cells were collected and injected into nude mice (*n* = 3/group). **(A)** Tumor volumes were measured at the indicated times; #*p* < 0.01 vs. scramble+si-NC, ^φ^*p* < 0.01 vs. anti-miR-224-3p+si-DHRS4-AS1. Tumors were collected **(B)** and measured **(C)** after injection on the 15th day. #*p* < 0.01 vs. scramble+si-NC, ^φ^*p* < 0.01 vs. anti-miR-224-3p+si-DHRS4-AS1. **(D,E)** Western blot detected stemness-related and EMT-related markers in xenograft tumors; the optical densities of proteins were determined with ImageJ software; #*p* < 0.01 vs. scramble+si-NC. All results are expressed as the mean ± SD.

The suppression of miR-224-3p significantly inhibited tumor volume and tumor weight (Figures 5A–C) *in vivo*. However, the knockdown of DHRS4-AS1 partially rescued xenograft tumor growth abrogation caused by anti-miR-224-3p (Figures 5A–C). Moreover, silencing DHRS4-AS1 partially reversed anti-miR-224-3p-mediated expression of cancer cell stemness-related markers and EMT-related genes in both mRNA and protein levels (Figures 5D,E). Compared with control group, OCT4, SOX2, CD34, and CD133 were decreased by silencing miR-224-3p, and downregulation of miR-224-3p also inhibited the expression of EMT-related factors, N-cadherin, ZEB1, and Vimentin in xenograft tumors. However, silencing miR-224-3p elevated E-cadherin expression in tumors (Figures 5D,E).

### Tumor Suppressors TP53 and TET1 Are Antagonistically Regulated by DHRS4-AS1 and miR-224-3p

**Figure 6:**
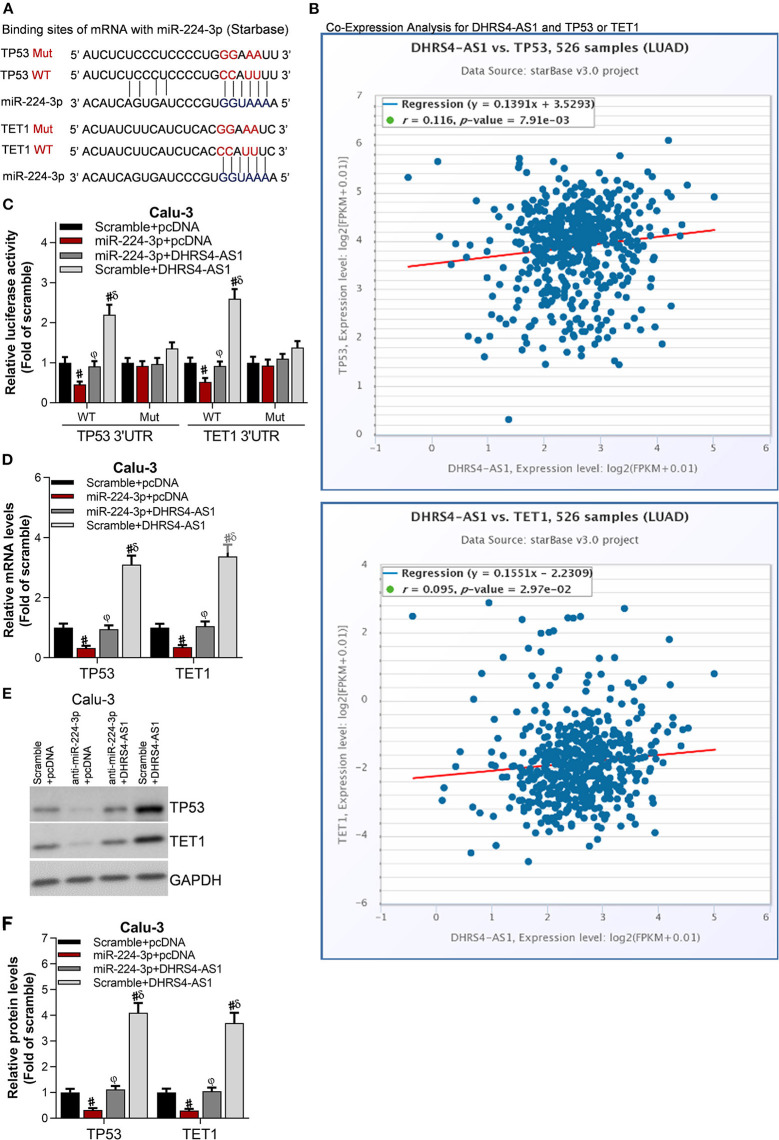
Tumor suppressors, TP53 and TET1 were antagonistically regulated by DHRS4-AS1 and miR-224-3p. **(A)** Schematic representation of the predicted miR-224-3p binding sites on TP53 and TET1 according to the StarBase prediction. **(B)** Co-expression analysis of DHRS4-AS1 with TP53 or TET1 according to the StarBase analysis. **(C)** Luciferase assay of pGLO-TP53-3′-UTR and pGLO-TET1-3′-UTR luciferase activity in transfected Calu-3 cells; #*p* < 0.01 vs. scramble+pcDNA, ^φ^*p* < 0.01 vs. miR-224-3p+pcDNA, ^δ^*p* < 0.01 vs. miR-224-3p+DHRS4-AS1. All data was normalized to wild type control group (Scramble+pcDNA) **(D)** Real-time PCR analysis of TP53 and TET1 expression in transfected Calu-3 cells; #*p* < 0.01 vs. scramble+pcDNA, ^φ^*p* < 0.01 vs. miR-224-3p+pcDNA, ^δ^*p* < 0.01 vs. miR-224-3p+DHRS4-AS1. **(E,F)** Western blot analysis of TP53 and TET1 expression in transfected Calu-3 cells; the optical densities of protein were determined with ImageJ software, #*p* < 0.01 vs. scramble+pcDNA, ^φ^*p* < 0.01 vs. miR-224-3p+pcDNA, ^δ^*p* < 0.01 vs. miR-224-3p+DHRS4-AS1. All data are expressed as the mean ± SD from three independent experiments, and each sample was repeated in triplicate.

The downstream molecules of DHRS4-AS1/miR-224-3p signaling were predicted in StarBase (Figure 6A). The most 10 potential targets were checked by RT-PCR (data not shown) and public starBase, TP53 and TET1 were proved to have positively expression with DHRS4-AS1, respectively (Figure 6B). When normalized to wildtype control group (WT Scramble+pcDNA), luciferase reporter assay shown that miR-224-3p significantly reduces the luciferase activity of TP53's 3′UTR and TET1's 3′UTR, and the DHRS4-AS1 totally rescues miR-224-3p-caused downregulated luciferase activity (Figure 6C). Consistently, miR-224-3p significantly downregulated the mRNA and protein levels of TP53 and TET1, which were increased by DHRS4-AS1 (Figures 6D–F).

## Discussion

Lung cancer has been listed as the leading cause of cancer-related death in humans. Cancer stem cells play an essential role in the aggressive and destructive behavior of lung cancer (Duma et al., 2019; Siegel et al., 2020). Cancer stem cells are positively associated with NSCLC recurrence, and these cells are closely connected with multidrug resistance and cancer initiation, proliferation, and differentiation (Heng et al., 2019). Importantly, lncRNAs have been reported to have important roles in NSCLC initiation, progression and stemness maintain by our and other groups (Zhan et al., 2017; Lu T. et al., 2018; Zhao et al., 2018; Huang et al., 2019; Li et al., 2020). AFAP1-AS1 promotes NSCLC cell proliferation and drug-resistance, correlates poor prognosis (Deng et al., 2015; Huang et al., 2019). TBILA, controlled by TGF-beta, promotes NSCLC progression *in vitro* and *in vivo* by cis-modulating HGAL and positively regulating S100A7/JAB1 signaling (Lu Z. et al., 2018). Linc00662 exerts its oncogenic functions by directly interacting with Lin28, a potential diagnostic and therapeutic target for patients with lung cancer (Gong et al., 2018). CASC11 maintains the cancer cell stemness of NSCLC by increasing TGF-beta1 expression (Fu et al., 2019). HAND2-AS1 suppresses NSCLC cells migration and invasion and contributes to cell stemness by interacting with TGF-beta1 (Miao et al., 2019). Consequently, exploring stemness-related lncRNAs as promising targets for the diagnosis and prognosis of NSCLC is necessary and meaningful. Here, high DHRS4-AS1 expression in NSCLC tumors indicated good clinical outcomes, and this lncRNA was found to down-regulated in NSCLC tumors, cancer cells and NSCLC-derived stem cell spheres. Furthermore, DHRS4-AS1 significantly inhibited the cancer stem cell colony formation ability and stemness of NSCLC cells. Our data also revealed that DHRS4-AS1 abrogates the expression of stemness markers, including OCT4, SOX2, CD34, and CD133, suppressed the expression of N-cadherin, ZEB1, and Vimentin, and increased E-cadherin expression in mRNA and protein levels. These findings suggest that DHRS4-AS1 may serve as an NSCLC tumor suppressor by inhibiting cancer cell stemness, and DHRS4-AS1 might be a promising target for NSCLC treatment.

Substantial evidence has reported that lncRNAs often function as miRNA sponges (Du et al., 2016; Furio-Tari et al., 2016; Song et al., 2019). Bioinformatics analysis and experiments data shown DHRS4-AS1 directly binds to miR-224-3p to regulate cancer cell stemness in NSCLC. Previous studies reported that miR-224-3p promotes breast cancer development by targeting FUT4 (Feng et al., 2016), promotes cervical cancer by repressing FIP200-mediated autophagy (Fang et al., 2016), and participates in the recurrence of human osteosarcoma (Xu et al., 2018). Nevertheless, the role of miR-224-3p in the stemness of NSCLC has not been explored. Here, miR-224-3p expression elevated in NSCLC tissues, cancer stem cells and cancer stem cell spheres. Furthermore, miR-224-3p knockdown inhibited cancer stem cell colony and sphere formation abilities. miR-224-3p decreased the expression of OCT4, SOX2, CD34, and CD133, and silencing miR-224-3p inhibited the expression of EMT-related factors, N-cadherin, ZEB1, and Vimentin but elevated E-cadherin expression *in vitro* and *in vivo*. DHRS4-AS1 acts as a tumor suppressor since it blocks the miR-224-3p-mediated silencing of TP53 and TET1, resulting in the inhibition of tumor growth *in vivo*. Our findings imply that the inhibition of miR-224-3p could improve NSCLC by suppressing cancer cell stemness.

Here, the interactions of RNAs (DHRS4-AS1 and miR-224-3p; miR-224-3p and 3′-UTRs of TP53 /TET1) was checked by luciferase assays developed by Clement et al. (2015). Our data revealed that miR-224-3p may target the 3′-untranslated regions (3′-UTRs) of TP53 and TET1, which are well known tumor suppressors in cancers, including lung cancer (Behan et al., 2019). Consistently, we observed that miR-224-3p can decrease the DHRS4-AS1 luciferase levels indicating that DHRS4-AS1 functions as ceRNA of miR-224-3p, and decreases it's regulation to downstream genes. In NSCLC cells, TP53 and TET1 were antagonistically regulated by miR-224-3p and DHRS4-AS1. All the luciferase data was normalized to wildtype control group (Scramble or Scramble+ pcDNA) to exclude endogenous mRNA or miRNA interruption as well as other group reported (Clement et al., 2015; Cheng et al., 2019; Hu et al., 2019; Zhang et al., 2019). These findings demonstrate that DHRS4-AS1/miR-224-3p signaling and downstream genes, TP53 and TET1 play important roles in the cancer cell stemness of NSCLC *in vitro* and *in vivo*.

In conclusion, DHRS4-AS1 functions as a tumor suppressor by reducing the cancer cell stemness of NSCLC, while miR-224-3p may serve as an oncogenic miRNA in NSCLC. The TP53- and TET1-associated DHRS4-AS1/miR-224-3p axis constitutes NSCLC progression by modulated cancer cell stemness *in vitro* and *in vivo*. DHRS4-AS1 and miR-224-3p could be considered potential therapeutic targets for NSCLC.

## Data Availability Statement

The original contributions presented in the study are included in the article/Supplementary Material, further inquiries can be directed to the corresponding author/s.

## Ethics Statement

The studies involving human participants were reviewed and approved by the Research Ethics Committee of the Affiliated Cancer Hospital of Nanjing Medical University. The patients/participants provided their written informed consent to participate in this study. The animal study was reviewed and approved by the Research Ethics Committee of Chengdu Medical College.

## Author Contributions

LS and YW: conceptualization and supervision. FY, WZ, and XX: methodology. FY, WZ, XX, and XL: validation. SL: formal analysis and resources. SL, YW, FY, and WZ: investigation. CL and SL: data. LS: writing—original draft preparation. LS, FY, and WZ: writing—review, editing, and funding acquisition. All authors: have read and agreed to the published version of the manuscript.

## Conflict of Interest

The authors declare that the research was conducted in the absence of any commercial or financial relationships that could be construed as a potential conflict of interest.
