# Supplementary material for: LncRNA DHRS4-AS1 Inhibits the Stemness of NSCLC Cells by Sponging miR-224-3p and Upregulating TP53 and TET1
Source: Front Cell Dev Biol. 2020 Dec 23;8:585251. doi: 10.3389/fcell.2020.585251 (PMC7786137; doi:10.3389/fcell.2020.585251)
Supplement: Supplementary file 1 [file Data_Sheet_1.PDF]

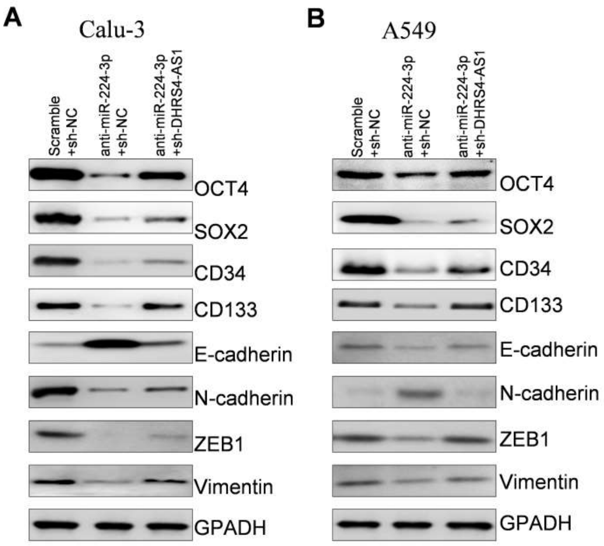

Supplementary figure 1: Western blot analysis of the expression of cancer stemness-related genes and EMT-related factors after 72 h of transfection.

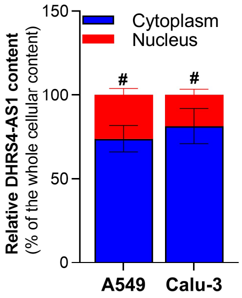

Supplementary figure 2: real-time PCR and nucleocytoplasmic separation assay analysis DHRS4-AS1 was mainly distributed in cytoplasm rather than in nucleus #p<0.01 versus cytoplasm group.
